# Tentative Identification of Phytochemicals from *Smilax glabra* and *Smilax corbularia* Extracts by LC-QTOF/MS and Their Bioactive Potential

**DOI:** 10.3390/plants11162089

**Published:** 2022-08-11

**Authors:** Peerapong Jeeno, Sukit Tongban, Pichamon Yana, Anurak Wongta, Kunrunya Sutan, Sumed Yadoung, Surat Hongsibsong

**Affiliations:** 1 School of Health Sciences Research, Research Institute for Health Sciences, Chiang Mai University, Chiang Mai 50200, Thailand; 2 Department of Chemistry, Faculty of Science and Technology, Chiang Mai Rajabhat University, Chiang Mai 50300, Thailand; 3 Environment, Occupational Health Sciences and Non-Communicable Disease Center of Excellence, Research Institute for Health Sciences, Chiang Mai University, Chiang Mai 50200, Thailand; 4 Environmental Science Program, Faculty of Science, Chiang Mai University, Chiang Mai 50200, Thailand

**Keywords:** antioxidant activity, anti-inflammatory activity, antibacterial activity, flavonoid, phenolic, LC-QTOF-MS

## Abstract

The Smilacaceae family has been used as a food source and herbal medicine for a long time. This study aims to identify the phytochemicals extracted from *Smilax glabra* and *Smilax corbularia* by using LC-QTOF/MS analysis and determine their bioactive potential. Compounds were identified from *S. glabra* and *S. corbularia* extracts by LC–QTOF-MS and it was found that longistylin A and CAY10435 have higher degrees of matching compounds (99.66% and 99.87%). *Smilax glabra* showed antioxidant capacity, i.e., DPPH and ABTS at percentage inhibitions of 71.94 ± 1.46% and 59.84 ± 4.80%, respectively, and FRAP at 730.69 ± 33.62 mg AAE/100 g sample. The total phenolic compound contents of the ethanol, methanol, and water extracts were 0.017 ± 0.001, 0.015 ± 0.001, and 0.016 ± 0.001 mg GAE/g, respectively, while the total flavonoid contents were 0.043 ± 0.002, 0.033 ± 0.002, and 0.006 ± 0.003 mg QE/g, respectively. The anti-inflammatory capacity showed 97.26% protection and 2.74% hemolysis. The antimicrobial activity can inhibit Gram-positive bacteria with a minimum inhibitory concentration (MIC) of 62.5 mg/mL and a minimum bactericidal concentration (MBC) of 500 mg/mL. *Smilax corbularia* showed antioxidant capacity, i.e., DPPH and ABTS at percentage inhibitions of 72.24 ± 0.64% and 39.87 ± 2.37%, respectively, and FRAP at 208.33 ± 50.80 mg AAE/100 g sample. The total phenolic compound contents of the ethanol, methanol, and water extracts were 0.006 ± 0.000, 0.007 ± 0.002, and 0.002 ± 0.001 mg GAE/g, respectively, while the total flavonoid contents of the ethanol and methanol extracts were 0.012 ± 0.001 and 0.008 ± 0.000 mg QE/g, respectively. The anti-inflammatory capacity showed 96.64% protection and 3.36% hemolysis. The antimicrobial activity of the extracts can inhibit Gram-positive bacteria with a MIC of 31.25 mg/mL and MBC of 125 mg/mL for the ethanol extract and a MIC of 125 mg/mL and MBC of 62.5 mg/mL for the methanol extract. In conclusion, *Smilax glabra* and *Smilax corbularia* were found to contain several phytochemicals that can be used for further study. Both Smilax species can also be used as sources of antioxidants and herbal medicines for killing Gram-positive bacteria.

## 1. Introduction

*Smilax glabra* Roxb. and *Smilax corbularia* Kunth. are herbal species in the Smilacaceae family and are Southeast Asian plants endemic to the area. They have infiltrated nearby species in China, Myanmar, Thailand, and Laos. In Thailand, they are commonly found in sparse woodlands, hilly evergreen forests, mixed deciduous forests, and deciduous dipterocarp forests in the northern part of the country and select places in the northeast. Both have unique characteristics that can be identified. *S. corbularia*’s head has dark red flesh, a delicate texture, and a buttery flavor, whereas *S. glabra*’s has white meat and a sweet and nutty flavor. In this scenario, rhizomes are used as drugs to treat syphilitic poisoned sores, the hypertonicity of limbs, eczema pruritus, carbuncle toxins, and many other ailments in traditional Chinese medicine. Furthermore, the subterranean component of *S. glabra* Roxb. can be incorporated into therapeutic soups, nutritional teas, and functional meals.

Smilacaceae has been identified to contain numerous biological activities, including antioxidant, anti-inflammatory, and antibacterial properties, according to contemporary pharmacological studies. Flavonoid and phenolic chemicals, according to research, are important active constituents in various therapeutic plants and foods. They have a wide variety of pharmacological effects [1]. Furthermore, cytokine production was used to test the anti-inflammatory activity of flavonoids [2,3]. *S. glabra* rhizome extract was tested for antibacterial activity and shown to inhibit Gram-positive bacteria [4]. According to investigations of DPPH radicals, ABTS radical scavenging, and reducing power [5], the antioxidant activity was attributed to its polyphenol components.

Astilbin is thought to have antibacterial, anticancer, anti-inflammatory, selective immunosuppressive, and antioxidant properties [6]. Antitumor effects were found in four more compounds, which were three astilbin stereoisomers known as neoastilbin, isoastilbin, and neoisoastilbin, as well as their aglycon taxifolin [7]. The bactericidal effects of four flavan-3-ols, including (–)–epicatechin and cinchonain Ib, were shown. Astilbin reduced the proliferation of LPS-stimulated mouse J774A.1 macrophage cells [8]. Epicatechin has also been linked to anti-inflammatory effects [9].

This study will investigate the antioxidant activity of *S. glabra* and *S. corbularia* extracts using DPPH, ABTS, and FRAP assays, as determined by the total phenolic compound and total flavonoid contents, anti-inflammatory action, and antibacterial activity. The quantitative dataset will be screened using LC-QTOF/MS to determine its main ingredients.

## 2. Results and Discussion

### 2.1. Characterization by LC-QTOF-MS

**Figure 1:**
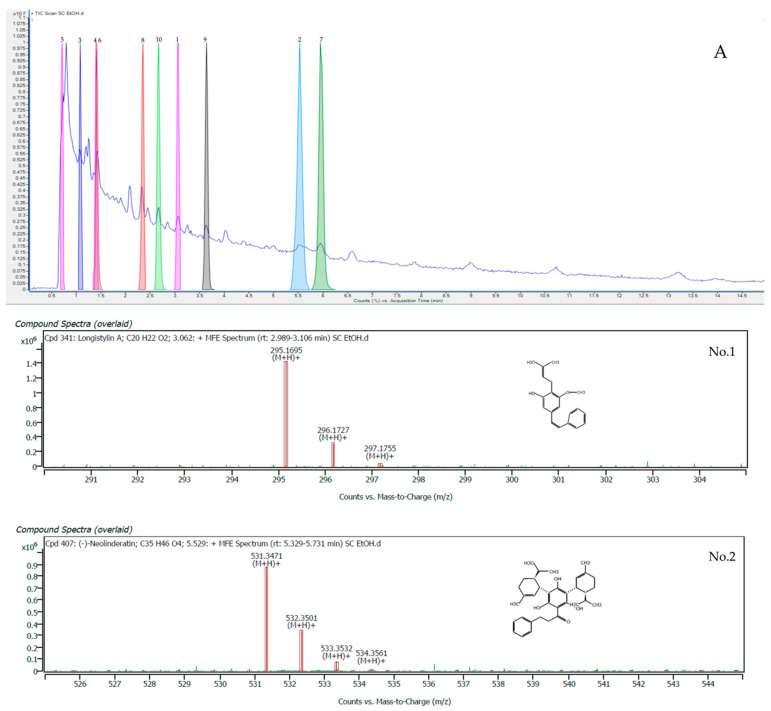

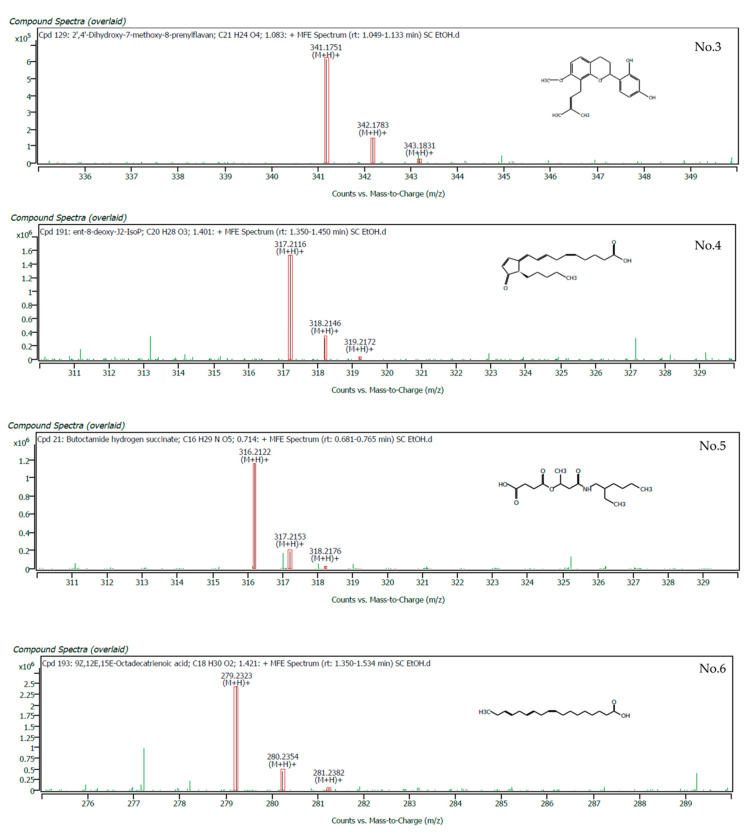

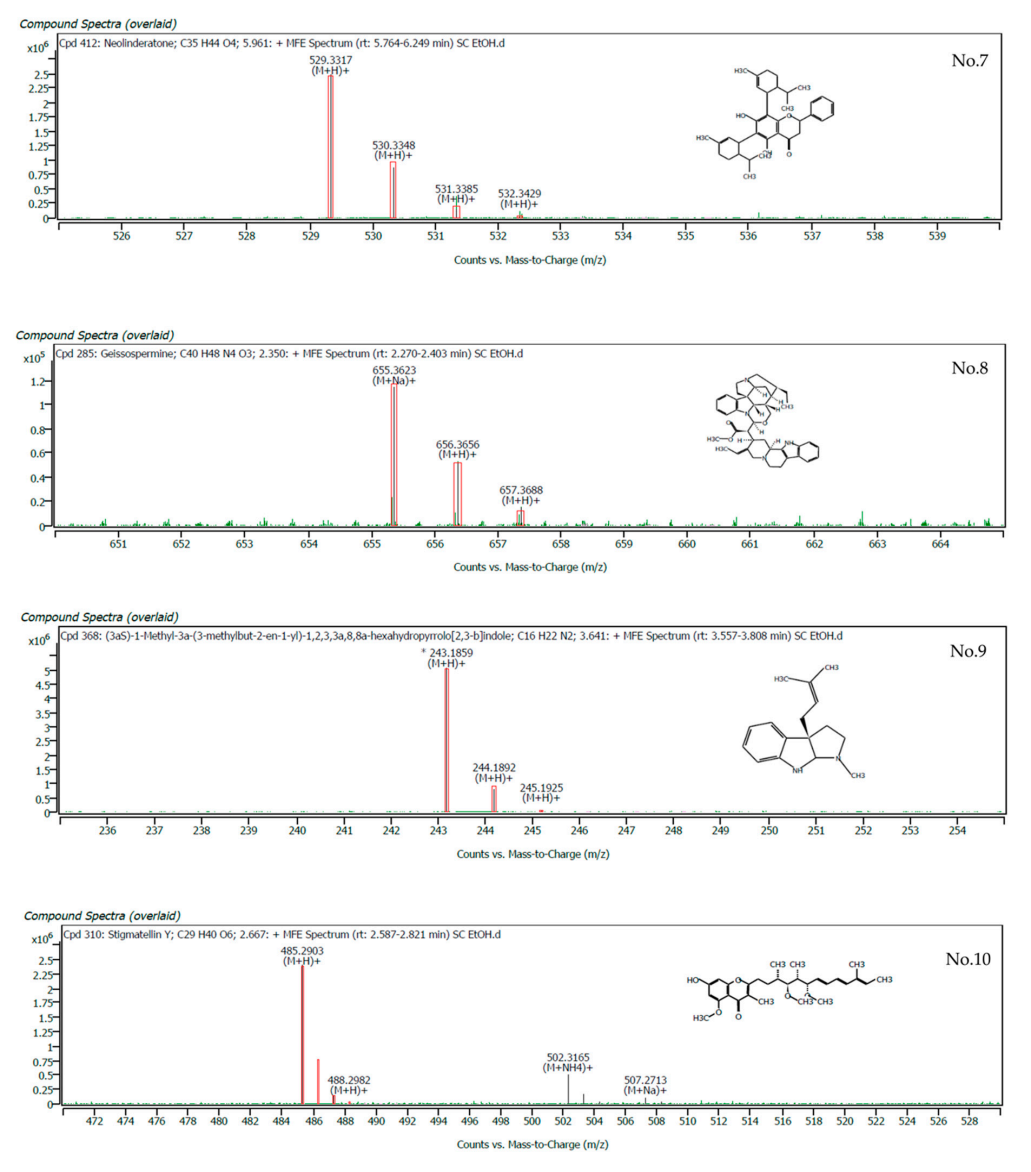
* LC-QTOF/MS analysis of the top ten compounds in terms of matching scores of *S. cobularia* ethanolic crude extract: (**A**) LC-OTOF-MS chromatogram; (**No. 1**) mass spectrum of longistylin A; (**No. 2**) (–)–neolinderatin; (**No. 3**) 2′,4′–dihydroxy–7–methoxy-8-prenylflavan; (**No. 4**) ent–8–deoxy-J2–IsoP; (**No. 5**) butoctamide hydrogen succinate; (**No. 6**) 9Z,12E,15E-octadecatrienoic acid; (**No. 7**) neolinderatone; (**No. 8**) geissospermine; (**No. 9**) (3aS)–1–methyl3a–(3–methylbut–2–en–1–yl)–1,2,3a,8,8ahexahydropyrrolo [2,3–b] indole; and (**No. 10**) stigmatellin Y.

The experimentally measured mass of each ion was evaluated against the exact mass of the molecular formula and the expected isotope pattern for that formula. These data were used to score the matching of the ions with the database by the MassHunter Qualitative Analysis Software find-by-formula search of the Agilent MassHunter METLIN Metabolomics Database. The top ten compounds in terms of matching scores of the ethanolic crude extract of *S. cobularia*, which have a higher matching scores (96.96–99.66%), are shown in Table 1, and the mass spectra showing specific peaks and their corresponding compounds are shown in Figure 1.

As a result, all of the compounds were identified as longistylin A, (–)–neolinderatin, 2′,4′–dihydroxy–7–methoxy–8–prenylflavan, ent–8–deoxy–J2–IsoP, butoctamide hydrogen succinate, 9Z,12E,15E–octadecatrienoic acid, neolinderatone, geissospermine, (3aS)–1–methyl–3a–(3–methylbut–2–en–1–yl)–1,2,3,3a,8,8ahexahydropyrrolo [2,3–b] indole, and stigmatellin Y. Longistylin A belongs to the class of organic compounds known as stilbenes, which are organic chemicals. Longistylin A was discovered to have antibacterial qualities and significant antibacterial activity against MRSA in research of its properties [10]. In a previous study, it was found that stilbenes were isolated from *S. glabra,* but different types showed *trans*–resveratrol, *trans*–piceid, and piceatannol. Stigmatellin Y is a chromone [11]. In a previous study, three new stigmatellins were found, namely, stigmatellic acid, iso-methoxy–stigmatellin A, and stigmatellin C (3, isolated as isomers), which were isolated from the myxobacterium *Vitiosangium cumulatum* MCy10943^T^ [12].

**Figure 2:**
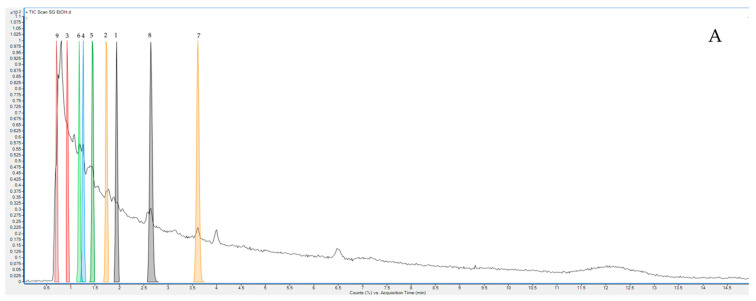

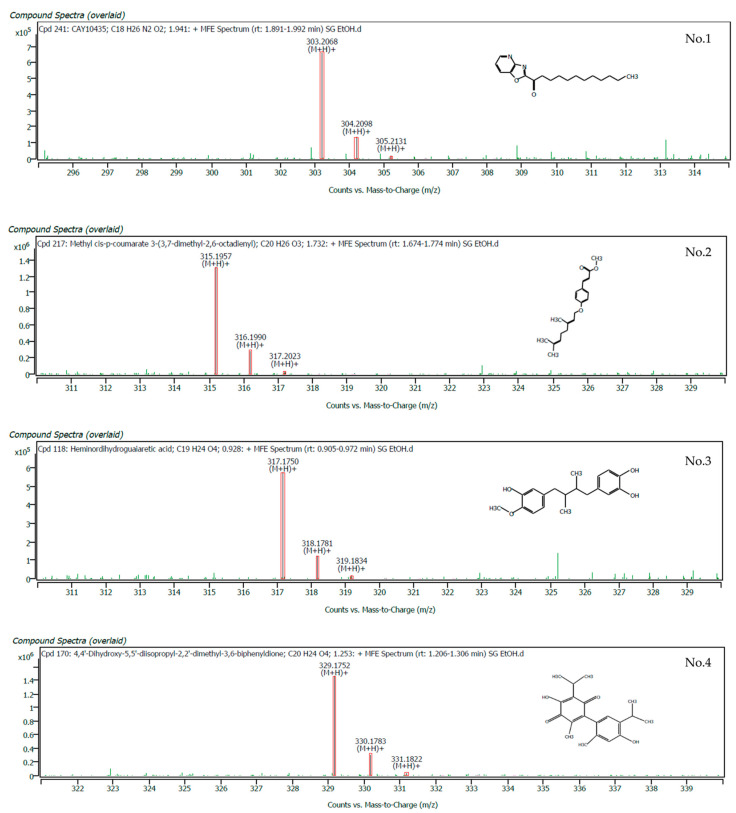

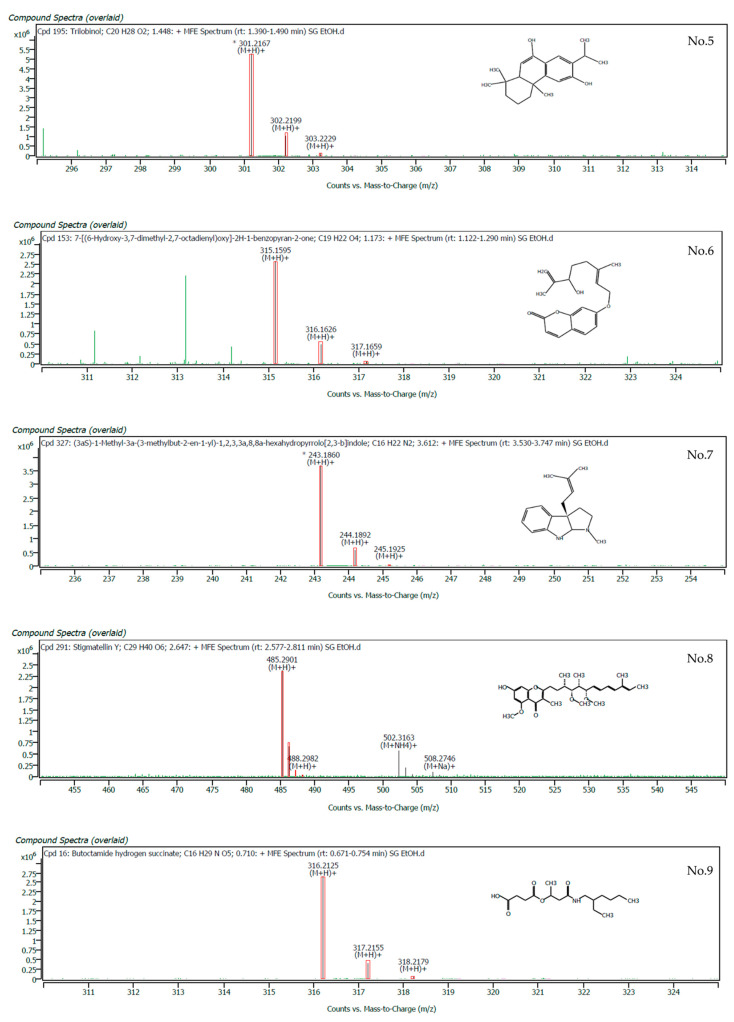
* LC-QTOF/MS analysis of the top ten compounds in terms of matching scores of *S. glabra* ethanolic crude extract: (**A**) LC-OTOF-MS chromatogram; (**No. 1**) mass spectrum of CAY10435; (**No. 2**) methyl cis–p–coumarate 3–(3,7–dimethyl–2,6–octadienyl); (**No. 3**) heminordihydroguaiaretic acid; (**No. 4**) 4,4′–dihydroxy–5,5′–diisopropyl–2,2′–dimethyl–3,6–biphenyldione; (**No. 5**) 8,11,14–eicosatriynoic acid; (**No. 6**) 7–[(6–hydroxy–3,7–dimethyl–2,7–octadienyl) oxy]–2H–1–benzopyran–2–one; (**No. 7**) (3aS)–1–methyl–3a–(3–methylbut–2–en–1–yl)–1,2,3,3a,8,8a–hexahydropyrrolo[2,3–b] indole; (**No. 8**) stigmatellin Y; and (**No. 9**) butoctamide hydrogen succinate.

The top nine compounds in terms of matching scores of the ethanolic crude extract of *S. glabra*, which have higher matching scores (96.29–99.87%), are shown in Table 2, and the mass spectra showing the specific peaks and their corresponding compounds are shown in Figure 2.

According to the results, all compounds were identified as CAY10435, methyl cis-p-coumarate 3–(3,7–dimethyl–2,6–octadienyl), hemihydro–guaiaretic acid, 4,4′–dihydroxy–5,5′–diisopro–pyl–2,2′–dimethyl–3,6–biphenyldione, 8,11,14–eicosatriynoic acid, 7–[(6-hydroxy–3,7–dimethyl–2,7–octadienyl)oxy]–2H–1–benzopyran–2–one, (3aS)–1–methyl–3a–(3–methylbut–2–en–1–yl)–1,2,3,3a,8,8a–hexahydropyrrolo [2,3–b] indole, stigmatellin Y, and butoctamide hydrogen succinate. From a medicinal chemistry standpoint, CAY10435 (oxazolopyridine) molecules have several benefits, and the antibacterial properties of oxazolopyridines were investigated [13]. In a previous study of the characterization of *Smilax glabra* by LC-QTOF/MS, six new phenolic compounds named smiglabrone A, smiglabrone B, smilachromanone, smiglastilbene, smiglactone, and smiglabrol were discovered. Smiglabrone A showed antimicrobial activity against *Canidia albicans*. Smilachromanone and smiglastilbene exhibited inhibitory activity against *Staphylococcus aureus* [11].

The (–)–Epicatechin, tran–stilbene, cis–stilbene oxide, cinchonain Ib, and neoastilbene were determined to be the active compounds shown in Table 3. According to previous phytochemical research, flavonoids are the main constituents, with flavanones being considerably more plentiful than other flavonoids [11]. These included the following: neoastilben, a flavonone, and (–)–epicatechin and cinchonain Ib, two flavans. (–)–epicatechin and cinchonain are bioactive compounds with antioxidant, antifungal, and antiviral properties.

### 2.2. Antioxidant Activity

To evaluate the antioxidant capacity, reducing power determination, as well as DPPH and ABTS radical scavenging activity, experiments were performed. Table 4 demonstrates that *S. corbularia* and *S. glabra* exhibited DPPH radical scavenging activity with percent inhibition values of 72.24 ± 0.64 (IC_50_: 583.06 mg/mL) and 71.94 ± 1.46 (IC_50_: 167.96 mg/mL), respectively. *S. corbularia* and *S. glabra* extracts had ABTS radical scavenging activity capacities of 59.84 ± 4.80 and 39.87 ± 2.37 percent inhibition, respectively. The percent inhibition values for *S. corbularia* and *S. glabra* extracts were significantly different, according to the *t*-test. The ferric ion reducing antioxidant power showed that *S. corbularia* had an antioxidant capacity of 208.33 ± 50.80 mg AAE/100 g sample, while *S. glabra* had an antioxidant capacity of 730.69 ± 33.62 mg AAE/100 g sample. Epicatechin, astilbin, neoastilbin, isoastilbin, and neo-isoastilbin at 50 g/mL displayed significant reducing power, with FRAP values of 499.33 ± 12.47, 148.22 ± 15.95, 223.78 ± 25.87, 400.44 ± 23.15, and 421.56 ± 4.16 M FeSO_4_ equivalent levels, respectively, in previous works [14]. The percent inhibition values for *S. corbularia* and *S. glabra* were significantly different according to the T test. The value for *S. corbularia* (IC_50_: 583.06 mg/mL) was higher than that of *S. glabra,* with a percent inhibition value of 71.94 ± 1.46 (IC_50_: 167.96 mg/mL), and a *t*-test revealed that the percent inhibition values of *S. corbularia* and *S. glabra* were significantly different. The DPPH in *S. glabra* extracted with ethanol was reported in a prior work at 32.3 µg/mL, which is a higher radical scavenging efficiency than the efficiency found in the current studies [15]. 

### 2.3. Total Phenolic Compound and Total Flavonoid Contents

The total phenolic compound (TPC) and total flavonoid (TFC) contents of *S. glabra* and *S. corbularia* are shown in Table 5. The ethanolic extract showed the maximum value for the TFC (0.043 ± 0.002 mg QE/g and 0.012 ± 0.001 mg QE/g, respectively), while the water extract showed the minimum value for the TFC for *S. glabra* (0.006 ± 0.003 mg QE/g) and was not detected in *S. corbularia.* In the present study, the total phenolic content of *S. glabra* was 0.017 ± 0.001 (mg GAE/g sample) in the ethanolic extract, which was higher than those of the methanolic extract and water extract. However, the total phenolic content of *S. corbularia* was 0.007 ± 0.002 (mg GAE/g sample) in the methanolic extract, which was higher than those in the ethanolic extract and water extract. One-way ANOVA indicated that the ethanol, methanol, and water fraction values were significantly different (*p* < 0.001 for the total flavonoid content, and *p* < 0.05 for the total phenolic compounds in the samples. However, for *Smilax glabra,* the extracts showed nonsignificant differences (*p* > 0.05). In previous reports, the total flavonoid and total phenolic contents of the ethanol extract of *S. glabra* were 420.26 ± 125.67 mg RE/g sample and 208.45 ± 3.74 (mg GAE/g sample), respectively [16]. In comparison, the total flavonoid and total phenolic contents of the 60% ethanol extract of *S. glabra* were 203.4 ± 9.1 mg RE/g sample and 262.6 ± 12.7 mg GAE/g sample, respectively [17].

### 2.4. Anti-Inflammatory Efficacy

The anti-inflammatory efficacy was measured by the suppression of hypotonicity-induced HRBC membrane lysis, i.e., the stabilization of the HRBC membrane. The percentage of membrane stabilization for ethanol stabilization was calculated at 100, 500, and 1000 µg/mL. Ethanolic extracts of *Smilax glabra* and *Smilax corbularia* are effective in inhibiting the heat-induced hemolysis of HRBC at different concentrations (100–1000 µg/mL), as shown in Table 6. It offered maximum protection of 97.26%, and hemolysis was 2.74% at 100 µg/mL in *Smilax glabra*; meanwhile, in *Smilax corbularia,* the maximum protection was 96.64%, and hemolysis was 3.36% at 100 µg/mL.

Inflammation is an essential contributor to various acute and chronic disease pathologies. A study of the acute oral toxicity of the aqueous crude extract of *Smilax glabra* in cytotoxicity research revealed that *Smilax glabra* had no toxicity impact on mice [18]. In another cytotoxic effect study, Gao et al. found that *Smilax glabra* inhibited the growth of HT-29 cell xenografts in a nude mouse model, and because the effects of *Smilax glabra* on body weight and lesions in the heart or liver were not observed, it was concluded that there was no toxic effect on mice [19]. Lipopolysaccharide (LPS)-induced inflammation in vitro promoted the release of inflammatory cytokines (IL-1β, IL-6, etc.) and activation of the nuclear factor-kappa B (NF-kB) pathway [2]. The lysosomal membranes that impact the inflammatory process are identical to the human red blood corpuscular membrane. Various illnesses are caused by the lysosomal enzymes generated during inflammation. The extracellular activity of these enzymes is linked to acute or chronic inflammation. Anti-inflammatory drugs work by inhibiting the cyclooxygenase enzyme, which is involved in the conversion of arachidonic acid to prostaglandins [20]. The study found that an ethanolic extract of *S. glabra* and *S. corbularia* had a membrane-stabilizing effect, suppressing hypotonicity-induced erythrocyte membrane lysis in a concentration-dependent manner. This action might be attributed to active components, such as flavonoids and triterpenoids. The impact of triterpenoids of the additional ethanol of Allium cepa (EEAC), which was shown to be concentration-dependent ligation, was a maximum protection of 95.18% at a concentration of 2000 µg/mL [21]. Additionally, it is known that Smilax contains much higher levels of astilbin.

### 2.5. Minimum Inhibitory Concentration and Minimum Bactericidal Concentration

**Figure 3:**
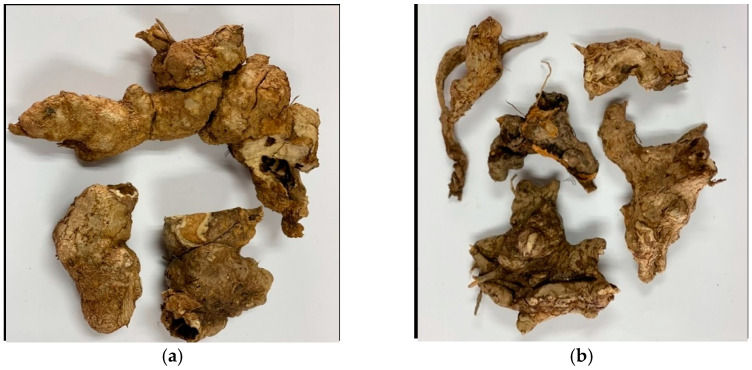
(**a**) *Smilax corbularia*; (**b**) *Smilax glabra*.

**Figure 4:**
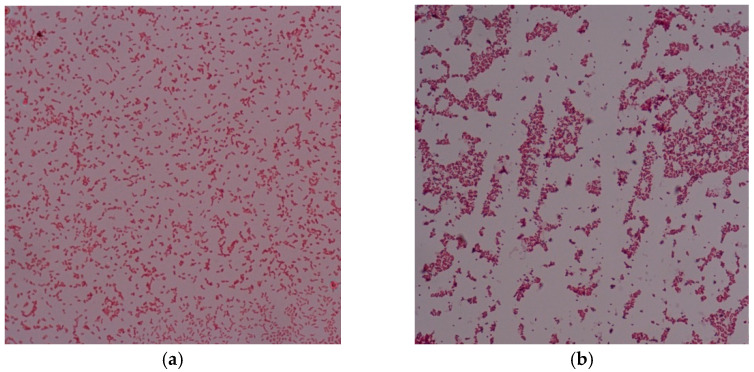
(**a**) Bacteria M1, negative bacteria and (**b**) bacteria M2, positive bacteria. Both bacteria were isolated from the mouth.

**Figure 5:**
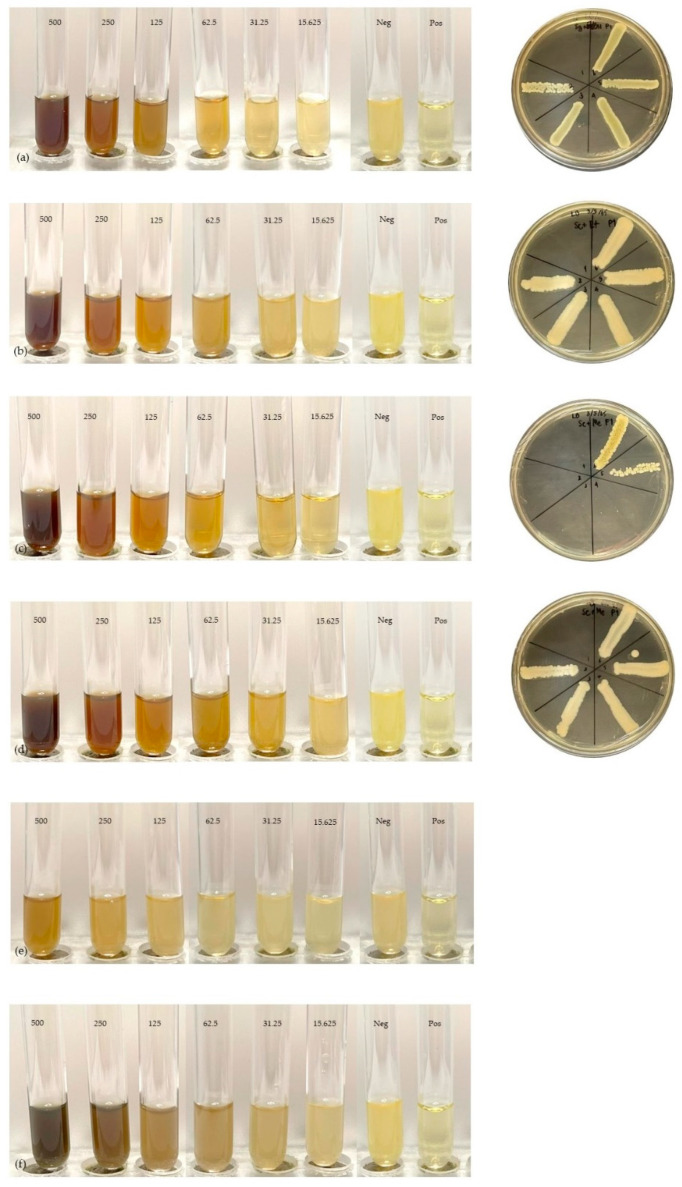
MIC and MBC with *Smilax corbularia*: (**a**) ethanol extract and bacteria M2. (**b**) Ethanol extract and bacteria M1. (**c**) Methanol extract and bacteria M2. (**d**) Methanol extract and bacteria M1. (**e**) Water extract and bacteria M2. (**f**) Water extract and bacteria M1.

**Figure 6:**
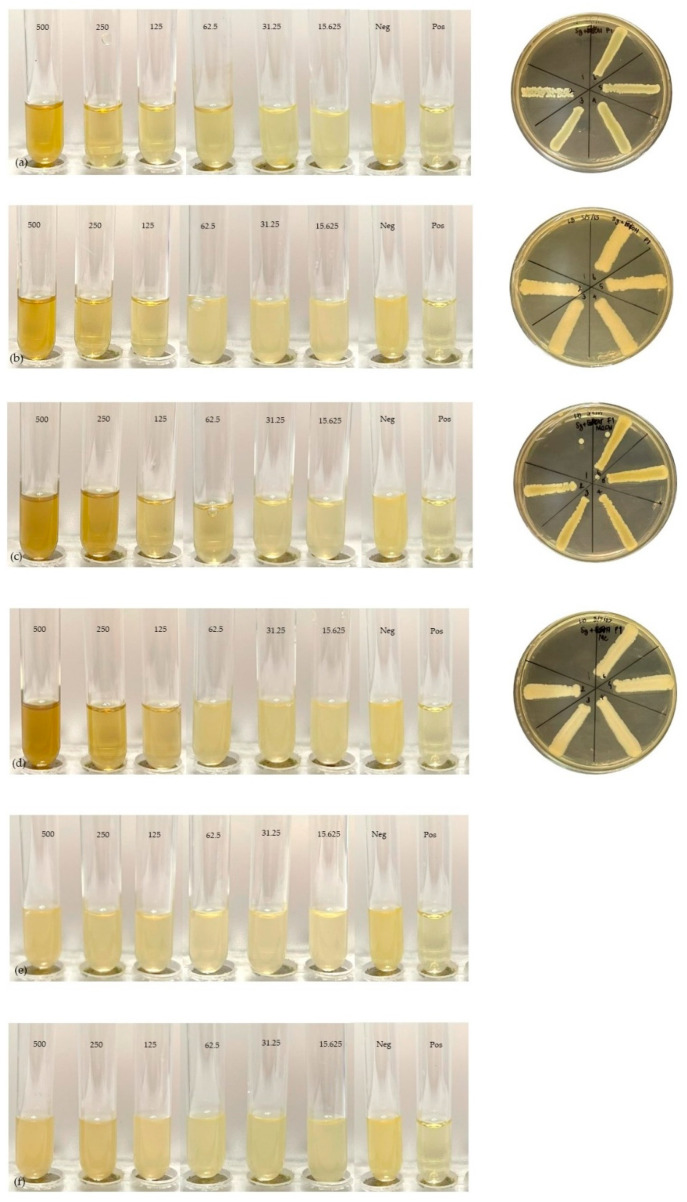
MIC and MBC with *Smilax glabra*: (**a**) ethanol extract and bacteria M2. (**b**) Ethanol extract and bacteria M1. (**c**) Methanol extract and bacteria M2. (**d**) Methanol extract and bacteria M1. (**e**) Water extract and bacteria M2. (**f**) Water extract and bacteria M1.

The MIC and MBC of *Smilax corbularia* and *Smilax glabra* (Figure 3) against the two strains (Figure 4) were measured for antibacterial activity (Table 7) and are shown in Figure 5 and Figure 6. The MIC values of *S. corbularia* from the ethanol extracted (EE) fraction and methanol extracted (ME) fraction showed activity against bacteria M2 compared with bacteria M1, with MIC values of 31.25 and 250 mg/mL, respectively, in the EE fraction, with MIC values of 125 and 250 mg/mL, respectively, in the ME fraction. The MIC values of *S. glabra* from the EE fraction and ME fraction showed activity against bacteria M2 compared with bacteria M1, with MIC values of 62.5 and 125 mg/mL, respectively, and the MBC values of *S. corbularia* from the EE fraction and ME fraction showed activity against bacteria M2 compared with bacteria M1, with MBC values of 125 and 500 mg/mL, respectively. The MBC values of *S. corbularia* from the ME fraction showed greater activity against bacteria M2 compared with bacteria M1, with MBC values of 62.5 and 500 mg/mL, respectively. The MBC values of *S. glabra* from the EE fraction and ME fraction showed activity against both bacteria, with MBC values of 500 mg/mL. Both bacteria were unaffected by the water extracted fraction. In a prior investigation, the minimal inhibitory concentration extracted from *Smilax macrophylla* leaves in 95% methanol demonstrated a substantial inhibitory effect against *S. aureus*, *E. coli*, and *B. subtilis* growth [22]. *Smilax China* L. polyphenols have antibacterial activity in the range of 195.31 to 781.25 μg/mL. *Smilax China* L. polyphenols demonstrated greater sensitivity in *Escherichia coli* and *Staphylococcus aureus* [23]. In previous studies, they found that seventeen compounds were antimicrobial and had antibacterial activity. (–)–Epicatechin, (+)-catechin, cinchonain Ib, and cinchonain Ia demonstrated antimicrobial properties [11].

## 3. Materials and Methods

### 3.1. Plant Collection

The 10 plants of each Smilax were collected from the BanSaluang Nok community forest, Maerim District, Chiang Mai, Thailand, which is located at 19°01′33.2″ N of the equator and 98°54′39.4″ E of the equator, on the date 2–3 October 2021.

### 3.2. Extraction of Plant Samples

Fresh *Smilax glabra* and *Smilax corbularia* were cleaned and dried in an oven at 50 °C for 1 h, which showed 75% and 68% water contents, respectively. Then, the dried samples were crushed into small pieces. One gram of crushed sample was extracted by using 30 mL of distilled water, ethanol (95%, JT Baker, NJ, USA), and methanol (99%, JT Baker, NJ, USA) and then soaked for 3 days at room temperature (25 °C). After that, the soaked sample was shaken at 2500 rpm for 5 min by using a shaker (OHAUS) and then filtered with Whatman No. 1. The filtered sample was evaporated to dryness by using a rotary evaporator (IKA model RV8, Staufen im Breisgau, Germany). The crude extract was weighed and redissolved in dimethyl sulfoxide (RCI Labscan, Bangkok, Thailand). The crude extract was stored in a refrigerator at 4 °C prior to analysis.

### 3.3. Characterization of Extract by LC-QTOF-MS

The qualitative dataset of the extracted sample was qualified by using LC-MS in tandem with a quadrupole time-of-flight mass spectrometer (QTOF-MS), according to the adopted method of Chiwat et al. (2021) [24]. In brief, the extracted samples were dissolved in 0.01% formic acid and ethanol (1:1, *v*/*v*) at a concentration of 1 mg/mL and cleaned up using a dispersive SPE kit with fat and pigments (Agilent Technology, Santa Clara, CA, USA). The sample solution was filled through a 0.22 μm filter. The qualitative dataset was determined using an Agilent 1290 Infinity II series coupled to a 6546 LC/Q-TOF instrument (Agilent Tech., Santa Clara, CA, USA), which consisted of a degasser, binary pump, column oven, and thermostat autosampler. The instrument settings were optimized as follows: LC conditions, UV at 330 nm; 0.2 mL/min flow rate; injection volume of 10 μL; and a mobile gradient system starting with 5% ACN and 95% water (1% formic acid), decreasing to 20% ACN in 5 min, 30% ACN in 5 min, 35% ACN in 5 min, 45% ACN in 5 min, 75% ACN in 5 min, and 95% ACN until the run ended. The chromatographic separation was accomplished using a ZORBAX Eclipse Plus C18 column (2.1 × 150 mm, 1.8 µm). The MS conditions involved an electrospray ionization (ESI) probe in the positive mode. The nebulizer was operated at 20 psi with a 7 L/min N_2_ flow. The capillary temperature was kept at 300 °C; the flow rate was set at 8 μL/min. The *m*/*z* range was 50–1000, the capillary voltage was 4500 V, and the dry heater temperature was 280 °C. The experimentally measured mass of each ion was evaluated against the exact mass of the molecular formula and the expected isotope pattern for that formula. These data were used to score the match of the ions with the database by the MassHunter Qualitative Analysis Software (Agilent MassHunter Workstation Qualitative Analysis version 10.0, California, USA) find-by-formula search of the Agilent MassHunter METLIN Metabolomics Database. The chemical parameters for compounds were determined using the Medlin library.

### 3.4. DPPH Radical Scavenging Activity

The free radical scavenging activity of the plant extract was determined using the method of Siok Peng Kek et al. (2017) [25]. In brief, the sample was analyzed using a 1,1-diphenyl-2-picryhydrazyl (DPPH) assay with minor modifications. The DPPH stock solution was prepared by dissolving 24 mg of DPPH in 100 mL of methanol. The absorbance of a working DPPH solution containing 10 mL of DPPH stock solution was adjusted to 1.1 at 517 nm, by diluting it with 45 mL of methanol. The DPPH working solution (100 µL) was mixed with 100 µL of plant extract solution (0.0156–1.000 mg/mL) in a 96-well plate. After incubation in the dark for 30 min, the absorbance was measured at 517 nm using a microtiter plate reader. The DPPH free radical scavenging activity was calculated as a percentage using the formula DPPH = [(A_control_ − A_sample_)/A_control_] × 100, where A_control_ and A_sample_ are the absorbance readings of the control and sample, respectively. The IC_50_ value for DPPH free radical scavenging activity corresponds to the sample concentration necessary to inhibit 50% of DPPH free radicals. The IC_50_ was determined graphically from the curve plot between the percentage of DPPH scavenging activity and sample concentration.

### 3.5. ABTS Radical Scavenging Activity

ABTS was used to determine the antioxidative potential based on the method described by Arnao et al. (2001) [26]. The ABTS reagent used 2,2-azino-bis(3-ethylbenzothiazoline-6-sulfonic acid) diammonium salt as a stable radical in an aqueous solution, and this solution was green. The absorbance measured at a wavelength of 734 nm is the value of the relationship between the percent inhibition of ABTS and sample concentration. ABTS radicals were produced by the reaction between 7 mM ABTS in water and 2.45 mM potassium persulfate and stored in the dark at room temperature for 12–16 h before use. The ABTS solution was then diluted with 80% ethanol to obtain an absorbance of 0.7 ± 0.02 at 734 nm. The ABTS working solution (190 µL) was added to a 96-well plate containing 10 µL of plant extract (0.0156–1.000 mg/mL). After incubation in the dark for 10 min, the absorbance was measured at 734 nm. An appropriate solvent blank was run in each assay. All measurements were carried out at least three times. The percent inhibition of absorbance at 734 nm was calculated using the formula ABTS radical scavenging effect (%) = [(A_ab_ − A_a_)/A_ab_] × 100, where A_ab_ is the absorbance of ABTS radical + methanol and A_a_ is the absorbance of ABTS radical + sample extract/standard. Trolox was used as a standard substance.

### 3.6. Ferric Ion Reducing Antioxidant Power Assay

The FRAP assay was performed using the method of Siok Peng Kek et al. (2017) [25]. In brief, 2.5 mL of 10 mM TPTZ (2,4,6-tripyridyl-s-trizine) solution in 40 mM HCl, 2.5 mL of 20 mM ferric chloride (FeCl_3_), and 25 mL of 300 mM acetate buffer at a pH of 3.6 were combined to make the FRAP reagent. Each 1 g of plant extract solution was diluted two times with distilled water, and aliquots of 10 μL of plant extract solution were combined with 190 µL of FRAP reagent on a 96-well microtiter plate. After 30 min in the dark, the absorbance of the sample was measured at 593 nm with a microtiter plate reader. Ascorbic acid was used for the standard curve (3.125–200 µg/mL), and FRAP was calculated against the standard curve. The result was expressed as milligrams of ascorbic acid equivalent per 100 g of plant extract (mg AAE/100 g).

### 3.7. Total Phenolic Compound Content

The total phenolic compound content was determined using the Folin–Ciocalteu method, which was modified by Dewanto et al. (2002) [27]. Gallic acid (Fluka, Switzerland) was used as the standard material in this method. As a result, the total phenolic content is given in milligrams of gallic acid per gram of sample weight. Gallic acid was used as the standard ingredient to produce the standard curve. Different concentrations of gallic acid of 0.02, 0.04, 0.08, 0.16, 0.32, and 0.64 mg/mL were obtained in 80% methanol by combining 12.5 μL of the sample and 12.5 μL of the Folin–Ciocalteau solution (MERCK, Germany) diluted 10 times with distilled water. After 6 min, 125 μL of 7% sodium carbonate (Na_2_CO_3_) and 100 μL of distilled water were added, and the mixture was allowed to react at room temperature for 90 min. After that, the absorbance was measured at 760 nm using a microplate reader (SPECTROstar^Nano^). A calibration curve was used to quantify the phenolic concentration. The results were given in milligrams of gallic acid equivalents per gram of sample (mg GAE/g sample).

### 3.8. Total Flavonoid Content

The total flavonoid content was measured by a colorimetric assay described previously by Miliauskas et al. (2004) [28], with slight modifications. A total of 25 μL of sample solution was placed into a 96-well plate, and 7.5 μL of 7% NaNO_2_ solution (LOBA CHEMIE PVT. Ltd.) and 12.5 μL of distilled water were added and mixed thoroughly. The solution was allowed to stand at room temperature for 5 min. Next, 15 μL of 10% AlCl_3_ solution (QRëC) was added to the flask, mixed well, and kept at room temperature for 5 min. Finally, 50 μL of 1 M NaOH solution (ACI Labscan) and 27.5 μL of distilled water were added, mixed well, and kept at room temperature for 5 min. Absorbance at 510 nm was measured against the water blank using a microplate reader (SPECTROstar^Nano^), and the concentration of flavonoids was estimated using calibration curves. The results were expressed as micrograms of quercetin equivalents per gram of sample (μg QE/g sample).

### 3.9. Anti-Inflammatory Activity

The method for stabilizing the membrane of human red blood cells (HRBC) was used [29].

For the plant sample solution, the sample was prepared at concentrations of 1000, 500, and 100 µg/mL in a buffer solution with a pH of 7.4.

For red blood cell preparation, Alsever solution (SIGMA-ALDRICH, Gillingham, UK) was added to the collected volunteers’ blood. The samples were centrifuged at 3000 rpm for 20 min at 4 °C with gentle shaking. Only red blood cells were collected, the NaCl solution was added, and the mixture was gently shaken. The samples were centrifuged again at 3000 rpm for 20 min at 4 °C. Only red blood cells were stored for analysis.

For preparation of 10% *v*/*v* HRBC, Add 9 mL of NaCl solution to 1 mL of washed red blood cells and then gently shake well.

For sample analysis, one milliliter of a buffer solution with a pH of 7.4 was pipetted into the test tube. Then add 2 mL of NaCl solution, 0.5 mL of 10% *v*/*v* HRBC, and 0.5 mL of sample solution in the test tube. Then, the mixture was gently shaken. The samples were incubated for 30 min at 37 °C in a water bath and then centrifuged at 3000 rpm for 10 min. The absorbance of the solution was measured at a wavelength of 560 nm. The control was 10% *v*/*v* HRBC solution + distilled water.

The calculation method was as follows:

% Protection = 100 − [(A_1_/A_0_) × 100]

where A_0_ = absorbance of control; A_1_ = absorbance of the sample.

% Hemolysis = [(A_1_/A_0_) × 100]

where A_0_ = absorbance of control; A_1_ = absorbance of the sample.

### 3.10. Antibacterial Activity

The minimum inhibitory concentration (MIC) and minimum bactericidal concentration (MBC) were determined using the method of Prashik Parvekar et al. (2022) [30]. In brief, by observing the visible growth of bacteria in the agar broth, the standard broth dilution method (CLSI M07-A8) was employed to investigate the antimicrobial effectiveness of the extracts. Serial twofold dilutions of *S. corburalia* and *S. glabra,* with adjusted bacterial concentrations from 500 mg/mL to 15.625 mg/mL (the turbidity was equal to 0.5 McFarland standard turbidity, which was approximately 1 × 10^8^ CFU/mL), were used to determine the MIC in LB broth. The control contained only inoculated broth with 1 mg/mL gentamycin and was incubated for 12 h at 37 °C. The extracted concentration at no visible growth in the tubes is known as the MIC endpoint. To confirm the MIC value, the visual turbidity of the tubes was measured both before and after incubation. Following the MIC analysis of the extracts, each tube was seeded on LB agar plates and incubated for 12 h at 37 °C. The MBC endpoint occurs when the bacterial population is eliminated at the lowest antimicrobial agent concentration. This was accomplished by looking for bacteria on pre- and postincubated agar plates.

## 4. Conclusions

*S. glabra* and *S. corbularia* extracted with ethanol, methanol and water have bioactive potential, can have antioxidant capacity and contain phenolic and flavonoid compounds. The antimicrobial results revealed that both samples inhibited Gram-positive bacteria more than Gram-negative bacteria. The anti-inflammatory results showed the highest protective effect, and LC-QTOF/MS analysis showed that it was due to stilbene longistyline A, CAY10435 (oxazolopyridine) and flavonoids such as (–)–neolinderatin and neolinderatone. *S. glabra* and *S. corbularia* might be exploited as possible sources for folk medicine for the discovery of novel products as antibacterial, anti-inflammatory, and antioxidant agents.

## Figures and Tables

**Table 1:** Compounds identified from the ethanolic crude extract of *S. cobularia* according to LC-QTOF-MS.

No.	Compound	Formula	RT	Matching Score (%)	*m*/*z*	Mass	Mass Diff (db/ppm)
1	Longistylin A	C_20_ H_22_ O_2_	3.062	99.66	295.1695	294.16219	0.7
2	(–)–Neolinderatin	C_35_ H_46_ O_4_	5.529	99.29	531.3471	530.33971	0.18
3	2′,4′-Dihydroxy-7-methoxy-8-prenylflavan	C_21_ H_24_ O_4_	1.083	99.03	341.17507	340.16781	1.04
4	ent-8-deoxy-J2-IsoP	C_20_ H_28_ O_3_	1.401	98.69	317.21156	316.2042	1.13
5	Butoctamide hydrogen succinate	C_16_ H_29_ N O_5_	0.714	98.59	316.21217	315.20487	0.94
6	9Z,12E,15E-Octadecatrienoic acid	C_18_ H_30_ O_2_	1.421	98.24	279.23231	278.22498	1.42
7	Neolinderatone	C_35_ H_44_ O_4_	5.961	97.94	529.33167	528.32435	0.73
8	Geissospermine	C_40_ H_48_ N_4_ O_3_	2.35	97.87	655.36233	632.37316	0.83
9	(3aS)-1-Methyl-3a-(3-methylbut-2-en-1-yl)-1,2,3,3a,8,8ahexahydropyrrolo [2,3-b] indole	C_16_ H_22_ N_2_	3.641	97.74	243.18592	242.17866	1.51
10	Stigmatellin Y	C_29_ H_40_ O_6_	2.667	96.96	485.29033	484.28292	0.89

**Table 2:** Compounds identified from the ethanolic extract of *S. glabra* crude according to LC-QTOF-MS.

No.	Compound	Structure	RT	Matching Score (%)	*m/z*	Mass	Mass Diff (db/ppm)
1	CAY10435	C_18_ H_26_ N_2_ O_2_	1.941	99.87	303.20681	302.19951	0.26
2	Methyl cis–p–coumarate 3–(3,7–dimethyl–2,6–octadienyl)	C_20_ H_26_ O_3_	1.732	99.61	315.19574	314.18845	0.82
3	Heminordihydroguaiaretic acid	C_19_ H_24_ O_4_	0.928	99.21	317.17504	316.16776	0.97
4	4,4′–Dihydroxy–5,5′–diisopropyl–2,2′–dimethyl–3,6–biphenyldione	C_20_ H_24_ O_4_	1.253	98.73	329.17517	328.16786	1.22
5	8,11,14–Eicosatriynoic acid	C_20_ H_28_ O_2_	1.448	97.91	301.21666	300.20935	1.38
6	7–[(6–Hydroxy–3,7–dimethyl–2,7–octadienyl) oxy]–2H–1–benzopyran–2–one	C_19_ H_22_ O_4_	1.173	97.85	315.15953	314.1522	1.26
7	(3aS)–1–Methyl–3a–(3–methylbut–2–en–1–yl)–1,2,3,3a,8,8a–hexahydropyrrolo [2,3–b] indole	C_16_ H_22_ N_2_	3.612	97.6	243.18597	242.1787	1.68
8	Stigmatellin Y	C_29_ H_40_ O_6_	2.647	97.23	485.29014	484.2827	0.58
9	Butoctamide hydrogen succinate	C_16_ H_29_ N O_5_	0.71	96.29	316.21251	315.20519	1.95

**Table 3:** Compounds in the library according to LC-QTOF-MS.

No.	Compound	Structure	RT	Mass	Mass Diff (db/ppm)
1	(–)–Epicatechin	C_15_H_14_O_6_	0.694	290.0766	−8.46
2	Tran–stilbene	C_14_H_12_	6.045	180.0939	−0.21
3	Cis–stilbene oxide	C_14_H_12_O	1.731	196.0888	−0.46
4	Cinchonain Ib	C_24_H_20_O_9_	1.313	452.1125	3.89
5	Neoastilbene	C_21_H_22_O_11_	0.711	450.1153	−2.00

**Table 4:** DPPH scavenging activity, ABTS radical scavenging activity, and ferric ion reducing antioxidant power of the ethanolic extracts of *Smilax glabra* and *Smilax corbularia*.

Sample	Method				
	DPPH-Scavenging Activity		ABTS Radical Scavenging Activity		Ferric Ion Reducing Antioxidant Power
	%Inhibition	IC_50_	%Inhibition	IC_50_	mg AAE/100 g Sample
*Smilax corbularia*	72.24 ± 0.64	583.06	39.87 ± 2.37	1662.37	208.33 ± 50.80
*Smilax glabra*	71.94 ± 1.46	167.96	59.84 ± 4.80	2540.34	730.69 ± 33.62
* p * value *	0.005		0.003		>0.001

* mean *p* value correspondingly compared with Smilax corbularia and Smilax graba by *t*-test.

**Table 5:** Total phenolic compound and total flavonoid contents of *Smilax glabra* and *Smilax corbularia*.

	Sample	Method			*p* Value
		Ethanol Extract	Methanol Extract	Water Extract	
Total phenolic compound (mg GAE/g)	*Smilax corbularia*	0.006 ± 0.000	0.007 ± 0.002	0.002 ± 0.001	0.002
	Smilax glabra *	0.017 ± 0.001	0.015 ± 0.001	0.016 ± 0.001	0.068
Total flavonoid content (mg QE/g)	*Smilax corbularia*	0.012 ± 0.001	0.008 ± 0.000	ND	<0.001
	*Smilax glabra*	0.043 ± 0.002	0.033 ± 0.002	0.006 ± 0.003	<0.001

Each value represents the mean ± SD (*n* = 3). *p* < 0.001 corresponded to each extracted total flavonoid content, and *p* < 0.05 corresponded to each extracted total phenolic compound content. * indicates *p* > 0.05 compared with each extracted total phenolic compound content, ND = not detected.

**Table 6:** Anti-inflammatory effects of the ethanolic extracts of *Smilax glabra* and *Smilax corbularia*.

Sample	Concentration (µg/mL)					
	1000		500		100	
	% Protection	% Hemolysis	% Protection	% Hemolysis	% Protection	% Hemolysis
*Smilax corbularia*	96.29	3.71	95.68	4.32	96.64	3.36
*Smilax glabra*	96.90	3.10	96.20	3.80	97.26	2.74

Each value represents the mean (*n* = 3). *p* > 0.05 means the compared concentrations were insignificantly different.

**Table 7:** Minimum inhibitory concentration (MIC) and minimum bactericidal concentration (MBC).

Sample	Bacteria	MIC and MBC (mg/mL)					
		Ethanol Extract (EE)		Methanol Extract (ME)		Water Extract (WE)	
		MIC	MBC	MIC	MBC	MIC	MBC
*Smilax corbularia*	M1	250	500	250	500	-	-
	M2	31.25	125	125	62.5	-	-
*Smilax glabra*	M1	125	500	125	500	-	-
	M2	62.5	500	62.5	500	-	-

## Data Availability

Not applicable.
